# Tuberculous Otomastoiditis: A Diagnostic Dilemma

**DOI:** 10.7759/cureus.71307

**Published:** 2024-10-12

**Authors:** Pui Mun Yee, Iylia Ajmal Othman

**Affiliations:** 1 Otolaryngology - Head and Neck Surgery, Kulliyyah (Faculty) of Medicine, International Islamic University Malaysia, Kuantan, MYS; 2 Otolaryngology - Head and Neck Surgery, Sultan Ahmad Shah Medical Centre @IIUM, Kuantan, MYS

**Keywords:** chronic otitis media(com), extrapulmonary tuberculosis (eptb), mastoiditis mimicker, primary extrapulmonary tuberculosis, tuberculosis mastoiditis, tuberculosis (tb)

## Abstract

Where tuberculous (TB) infection is prevalent, the diagnosis of TB otomastoiditis (TOM) should be considered in a chronically discharging ear that does not respond to standard medical treatment.

We are reporting a case of TB otomastoiditis with an adjacent deep neck abscess in a healthy 18-year-old male. He presented with a five five-month history of right otorrhea with hearing loss and a concurrent right level two neck swelling, without any signs of acute infection. Aural polyp was seen occupying the external ear canal (EAC) obscuring the tympanic membrane. There was no clinical improvement despite oral, topical, and intravenous antimicrobial therapy. The audiogram showed right moderate to severe mixed hearing loss. Erythrocyte sedimentation rate (ESR) and Mantoux were positive; however, initial pus swab culture yielded Pseudomonas aeruginosa and was negative for acid-fast Bacilli (AFB). High-resolution computed tomography (HRCT) of the temporal bone showed multifocal bony erosion with soft tissue density occupying the EAC, middle ear, and mastoid air cells. Bezold and Citelli abscesses were also noted adjacent to the mastoid tip with an eroded outer cortex. The patient underwent mastoid exploration to obtain tissue for diagnosis and to clear the disease. The diagnosis of TB otomastoiditis was made based on intraoperative findings of caseous necrosis, which was culture positive for Mycobacterium tuberculosis but negative on AFB stain. Complete resolution of the disease was seen after three months of anti-TB treatment. His right hearing remains poor, thus he was counseled for a bone conduction hearing amplification device.

High clinical suspicion and early HRCT will expedite the delivery of treatment for suspected TB otomastoiditis. In some cases, surgical intervention is needed to obtain tissue for diagnosis, remove the sequestrum, and when there is clinical evidence of complications.

## Introduction

Tuberculosis (TB) is a chronic granulomatous infection caused by Mycobacterium tuberculosis. TB was the leading cause of death from a single infectious pathogen prior to the coronavirus (COVID-19) pandemic era [1]. Most cases were reported from South-East Asia countries (43%) [1]. Incidence of extrapulmonary TB is 15% in healthy individuals [1]. The middle ear and the larynx remain the most frequent sites of TB infection in the head and neck region, nevertheless, the highest mortality rate is seen when the infection involves the central nervous system (CNS) [2]. The involvement of both the middle ear and mastoid bone is termed tuberculous otomastoiditis (TOM), which is uncommon and accounts for 0.05%-0.9% of chronic middle ear infections [3]. Due to the rarity of the disease, available guidelines on TB do not have specific recommendations on TOM. Rather, it is discussed under extrapulmonary TB. Signs and symptoms of TOM are similar to chronic otitis media with or without cholesteatoma but not responsive to antimicrobials, leading to diagnostic delays [4]. Reported facial nerve palsy was more common in children [5]. Since it is associated with significant morbidities, such as facial paralysis, cochlear involvement, labyrinthitis, and intracranial abscess, a high index of suspicion is required to prevent complications. These complications resemble those of chronic otitis media, with or without cholesteatoma, but tend to occur over a more protracted course.

## Case presentation

**Figure 1:**
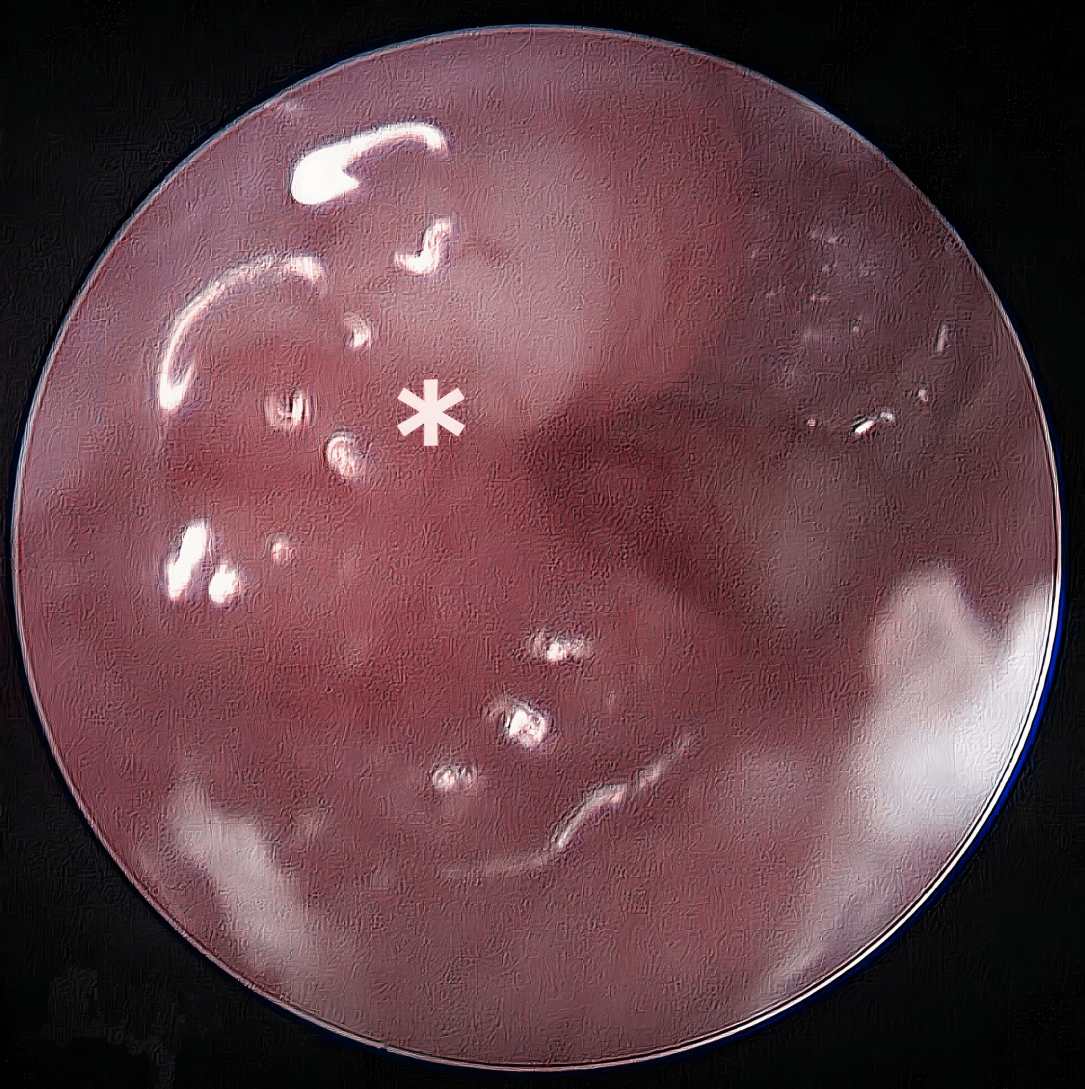
Otoendoscopy Extensive polyp (*) seen occupying the external ear canal and occluding the tympanic membrane with pus

An 18-year-old male presented with five months of recalcitrant, purulent right otorrhea with worsening hearing loss and right posterior neck swelling. Further history revealed positive close-contact history receiving anti-TB for TB spine. Examination revealed a friable polyp in the right ear external auditory canal (EAC) (Figure 1), totally obscuring the tympanic membrane (TM) with a 2 x 2 cm non-tender fixed firm mass adjacent to the right mastoid tip without overlying inflammatory changes. Audiometry showed right moderate to severe mixed hearing loss.

Cultures from the right EAC grew Pseudomonas aeruginosa, negative for AFB. Despite completing multiple courses of various oral, topical, and intravenous antibiotics, the patient's condition remains static with persistent otorrhea and hearing loss of a similar degree.

**Figure 2:**
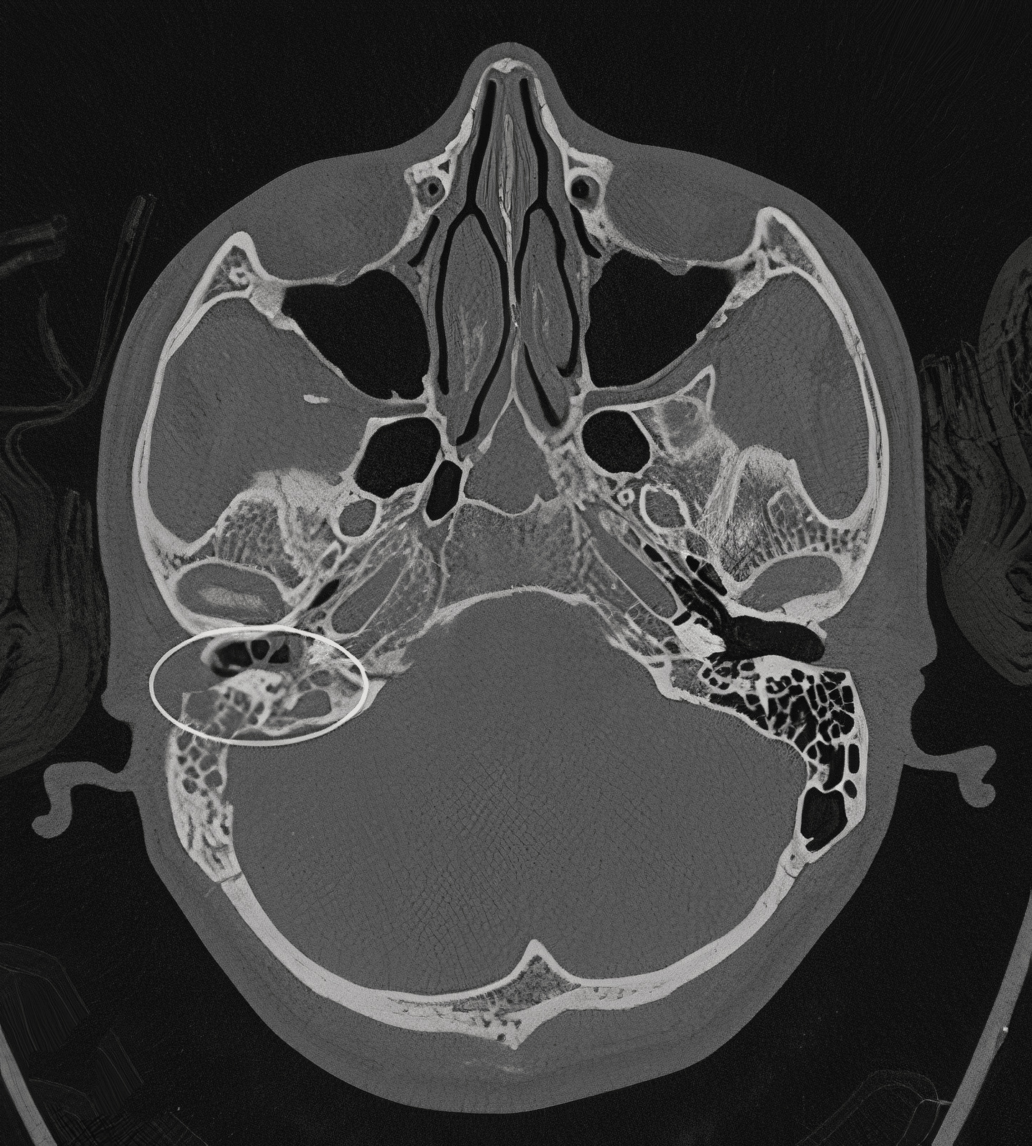
HRCT temporal (bone window, axial view) A right diploid mastoid with a soft-tissue-filled mastoid in the middle ear and external ear canal (circle). The left mastoid is clear. HRCT: high-resolution computed tomography

**Figure 3:**
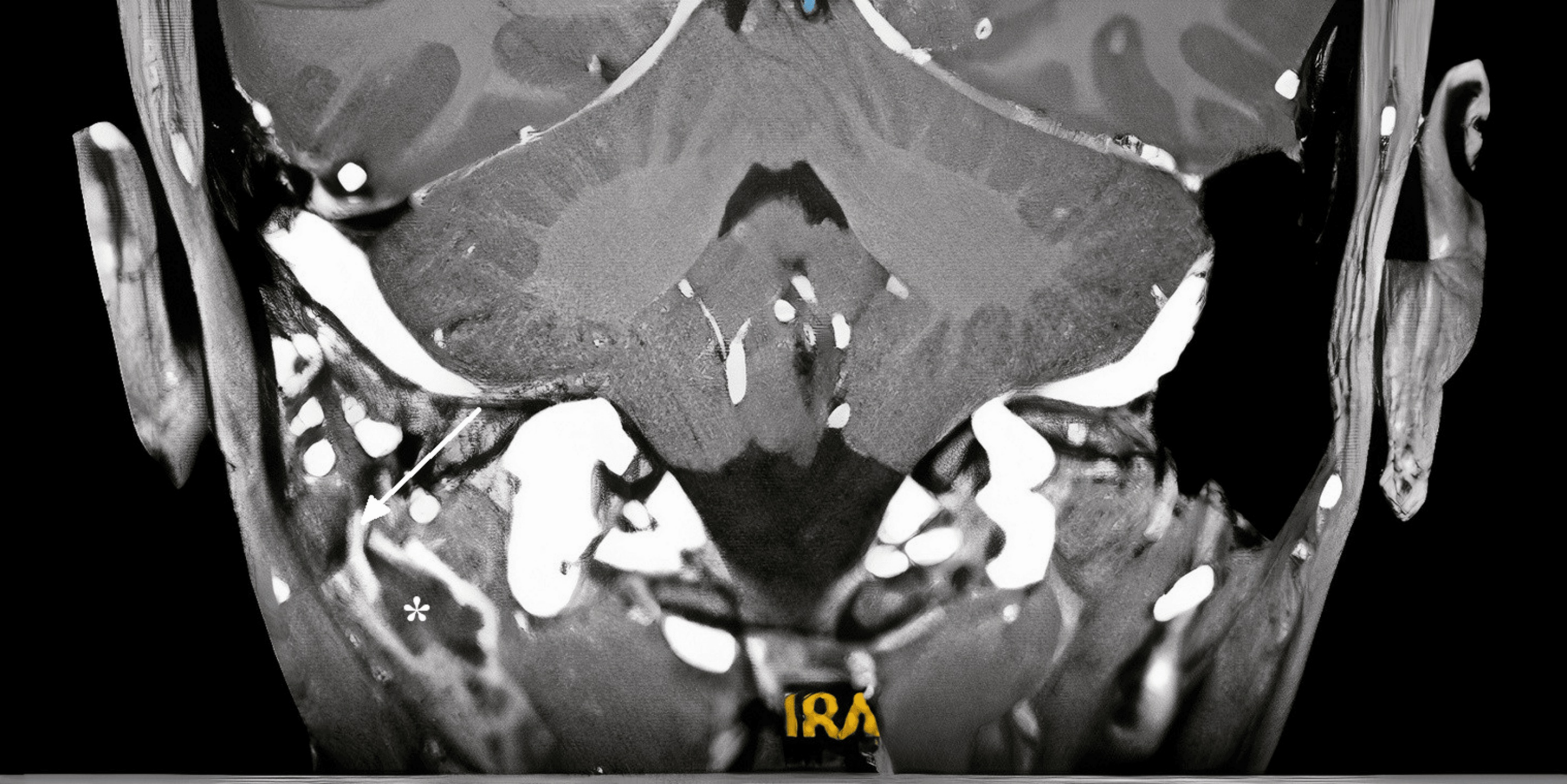
Contrast-enhanced MRI neck (T1 weighted, coronal view) Rim-enhancing lesion (*) over the right deep neck muscle adjacent to the eroded mastoid tip (arrow)

High-resolution computed tomography (HRCT) of the temporal bone showed multifocal bony erosion with soft tissue density occupying the EAC, middle ear, and mastoid air cells (Figure 2). Bezold and Citelli abscesses were also noted adjacent to the eroded outer cortex of the mastoid tip, consistent with magnetic resonance imaging (MRI) of the neck (Figure 3).

Given the history of chronic refractory disease and history of TB contact, we investigated the patient for possible TOM despite the initial negative AFB stain. His chest radiograph and sputum AFB were negative for TB. Fine-needle aspiration of the right neck swelling yielded a granulomatous inflammation, which was again negative for AFB. However, he had a raised erythrocyte sedimentation rate (ESR) and a positive tuberculin skin test.

The patient subsequently underwent modified radical mastoidectomy; primarily for tissue diagnosis with the secondary aim of providing middle ear aeration. Intraoperatively, the tympanic membrane appeared thickened with a pinpoint perforation, and the posterior EAC was eroded. The mastoid bone was sclerotic, with extensive granulation tissue with caseous necrotic material occupying the mastoid air cells, aditus, and middle ear cleft encasing an intact ossicular chain. These tissues were negative for AFB; however, on histology, a caseating granuloma was found, which subsequently grew rifampicin-sensitive Mycobacterium tuberculosis.

Based on intraoperative, histological, and microbiological results, the diagnosis of TOM was finally made. He was then started on anti-TB chemotherapy according to WHO and the standard local regime for drug-sensitive TB. This consists of a two-month intensive phase with ethambutol (E), isoniazid (H), rifampicin (R), and pyrazinamide (Z) followed by seven months of isoniazid (H) and rifampicin (R) [1].

Clinical resolution of the ear and neck disease was seen after three months of anti-TB treatment. His right hearing remains poor; thus, he was counseled for a contralateral routing of signals (CROS) hearing aid or a bone conduction hearing amplification device.

## Discussion

TOM is an extremely rare disease that mimics chronic otitis media with or without cholesteatoma clinically and radiological, delaying the diagnosis. Its classical clinical features include painless otorrhea, multiple tympanic membrane perforations, granulation tissue in the middle ear and mastoid, progressive conductive hearing loss, and facial nerve palsy [6]. Based on reports by Cavallin and Munoz [7], extensive bone destruction on CT without clinical signs of aggressive infection is suggestive of TOM.

Proposed mechanisms include eustachian tube insufflation, hematogenous spread, contiguous spread from primary foci, and direct inoculation from a perforated tympanic membrane [8]. In diagnosing TOM, a high index of suspicion is required, considering the clinical presentation, coexisting pulmonary lesion, history of TB contact, imaging, and bacteriological culture, as findings are often indistinct and non-specific. Only 50% of TOM is concurrent with pulmonary TB [9]. In our patient, routine screening for pulmonary TB was negative; however, he gave a history of TB contact with his aunt who is receiving treatment for TB spine. To date, there are no studies on TB contact along with TOM.

It was reported that TOM presents like chronic otitis media with or without cholesteatoma, often leading to a delayed diagnosis. Like our patient, refractory otorrhea with exuberant granulation tissue is a congruent finding across literature, providing a hint toward diagnosis [10]. Other clinical features reported include intact or thickened tympanic membrane, perforation of tympanic membrane which might be single or multiple, painful or painless refractory otorrhea, or conductive or sensorineural hearing loss of various degrees. The presence of caseous material with granulation tissue might be misdiagnosed as cholesteatoma [11]. Occasionally, a preauricular fistula, lymphadenopathy, facial nerve palsy, or labyrinthine fistula is found while a retroauricular fistula might develop postoperatively. 

Imaging is required not as a tool in establishing a diagnosis but as an aid in treatment planning and assessing the extent of the disease. Like chronic otitis media, HRCT of the temporal bone is the preferred imaging modality for evaluating the extent of disease in TOM due to its higher sensitivity in the presence of soft tissue and bony erosion [11,12] as well as the presence of otological complications, more accurately than MRI [13]. In both conditions, soft tissue is present in the middle ear; however, in TOM, the soft tissue is extensive and persists despite multiple courses of antibiotics, and the scutum is usually preserved [4,14]. The HRCT findings reported in the literature vary widely, which include varying degrees of mastoid sclerosis or preservation, cochlear fistula, bony erosion of the mastoid, tegmen, ossicles, facial canal, or otic capsule. In our case, there was sclerosis of the mastoid with posterior wall erosion of the external ear canal and mastoid tip, leading to the development of concurrent Bezold and Citelli abscesses. Despite the extensive soft tissue in the middle ear, there was no fistulisation of the otic capsule or erosion of the ossicles, tegmen, or scutum. The role of MRI in TOM is primarily limited to excluding intracranial extension.

Definitive diagnosis requires demonstration of Mycobacterium tuberculosis. Although staining for acid-fast Bacilli (AFB) is a rapid method, TOM frequently has low bacillus concentration [15] owing to its fastidious nature, which is further reduced with the usage of aminoglycoside ear drops, and a repeated swab will only improve detection by 50% [16]. Currently, TB culture using liquid media is the gold standard for the diagnosis of TB according to WHO; however, it is expensive and the average turnaround time for culture positivity alone is long (~20 days) [17]. Yaniv et al. also found most cases were superimposed with other infections, which might mislead the initial diagnosis in smear and culture-negative cases. This was similarly seen in our case, as his ear swab grew Pseudomonas aeruginosa and AFB was negative, thus masking the actual diagnosis. However, as clinical and imaging findings were disproportionate (i.e. the patient has relatively mild symptoms but there was extensive disease on clinical examination and HRCT temporal), suspicion of TOM remains high. In line with the Malaysian clinical practice guidelines on extrapulmonary TB, WHO suggested using molecular WHO-recommended rapid detection methods (mWRD), such as Xpert MTB/ RIF or Xpert MTB/RIF Ultra, for diagnosis, as it provides rapid detection (within two hours) and detects resistance to rifampicin; however TB culture and susceptibility is still required to monitor response to treatment and to exclude non-tuberculous mycobacterium (NTB) [8]. The Joint Committee of the American Thoracic Society/Infectious Disease Society of America/Centre of Disease Control and Prevention suggested a combination of tests (microbiological, histological, and molecular), as they lack sensitivity on their own [18]. Future guidelines or research on the diagnosis of TOM would prove invaluable in expediting diagnosis and treatment. In patients with difficulty in obtaining samples, surgery might be indicated to obtain tissue to expedite diagnosis, remove the sequestrum, and improve middle ear aeration. In our patient, the presence of caseous material was a giveaway in diagnosing TOM. However, due to granulation tissue encasing ossicles, the ossicles were sacrificed and planned for later reconstruction and hearing aid after disease clearance. Canal wall down mastoidectomy was opted for in our case for concurrent tissue diagnosis to eradicate extensive disease with posterior ear canal erosion. In certain cases, a combination of surgery and anti-TB is recommended to eradicate the disease. A delay in the initiation of anti-TB treatment might result in wound dehiscence, fistula formation, and tympanoplasty failure [19]. When facial nerve palsy is present, the prognosis depends on the early initiation of anti-TB treatment [20].

According to local guidelines, TOM should be treated according to extrapulmonary TB involving the bone with anti-TB drugs given for six to nine months (2EHRZ/4-7HR), consisting of a two-month intensive phase with ethambutol (E), isoniazid (H), rifampicin (R), and pyrazinamide (Z) followed by four to seven months of isoniazid (H) and rifampicin (R) [1]. Gradual response was noted upon initiation of anti-TB drugs; the ear was dry within six weeks and granulation tissue in the mastoid bowl regressed. The postauricular wound healed completely and the Bezold and Citelli cold abscesses resolved clinically with size reduction on follow-up HRCT temporal and neck. He is still under regular follow-up and planned for CROS or a bone conduction hearing aid as hearing rehabilitation.

## Conclusions

TOM is a rare, chronic middle ear disease thus there is often a delay in making the diagnosis. As TB is prevalent in Malaysia and Southeast Asia, the diagnosis of TOM should always be considered in a chronically discharging ear that does not respond to standard medical treatment. High clinical suspicion and early HRCT will expedite the delivery of treatment for suspected TOM and prevent complications. Diagnosis is confirmed with the demonstration of Mycobacterium tuberculosis; however, due to low yield, repeated sampling might be required. Although the molecular diagnostic test is rapid and able to detect a rifampicin-resistant strain, culture is still required to monitor response to treatment and exclude NTB. In some cases, surgical intervention is needed to obtain tissue for diagnosis, remove the sequestrum, and when there is clinical evidence of complications.
